# Exploring the Overlap of MASLD and IBD: Insights from a Single-Center Experience

**DOI:** 10.3390/ijms262110288

**Published:** 2025-10-22

**Authors:** Ana Stemate, Delia-Ionela Negru-Vodă, Ana Maria Patricia Mazurencu-Pele, Remus-Florin Popescu, Teodora-Iulia Spătaru, Lucian Negreanu

**Affiliations:** 1 “Carol Davila” University of Medicine and Pharmacy, Faculty of General Medicine, Dionisie Lupu Street, No. 37, Sector 2, 020021 Bucharest, Romania; patriciamazurencu@gmail.com (A.M.P.M.-P.); remus-florin.popescu@drd.umfcd.ro (R.-F.P.); teodora.spataru@drd.umfcd.ro (T.-I.S.); lucian.negreanu@umfcd.ro (L.N.); 2 Department of Gastroenterology 1, Emergency University Hospital, Splaiul Independenței Street, No. 169, Sector 6, 050098 Bucharest, Romania; annysto@yahoo.com

**Keywords:** metabolic dysfunction-associated steatotic liver disease (MASLD), liver fibrosis, liver steatosis, inflammatory bowel disease (IBD)

## Abstract

Metabolic dysfunction-associated steatotic liver disease (MASLD) is an increasingly prevalent condition worldwide, occurring both independently and, more importantly, as the most common extraintestinal complication of inflammatory bowel disease (IBD). The study primarily investigated MASLD prevalence in Crohn’s disease (CD) and ulcerative colitis (UC) to enhance prevention and early detection, especially given the recent approval of the first treatment for this condition. Secondary objectives included identifying risk factors, exploring the uric acid/high-density lipoprotein cholesterol ratio (UHRatio) as a steatosis marker and evaluating correlations between non-invasive fibrosis scores, Fibrosis-4 (FIB-4), AST-to-Platelet Ratio Index (APRI), and histological fibrosis severity. We conducted a prospective study on 58 patients diagnosed with IBD. The type of IBD was not independently associated with liver steatosis or fibrosis. Disease activity correlated significantly with hepatic steatosis in CD patients and with hepatic fibrosis in UC patients. The UHRatio proved useful in assessing steatosis prevalence, whereas FIB-4 and APRI scores did not correlate significantly with fibrosis severity. This study is, to our knowledge, the first to evaluate UHRatio as a potential predictor of MASLD in patients with IBD, expanding on previous findings reported in the general population. Our results suggest that this non-invasive biomarker, previously used to identify MASLD, may improve early prediction and could serve as a useful screening tool for MASLD in IBD patients.

## 1. Introduction

Inflammatory bowel disease (IBD), represented by Crohn’s disease (CD) and ulcerative colitis (UC), are chronic, non-infectious inflammatory disorders that can affect the entire gastrointestinal tract. Up to 50% of patients present systemic extraintestinal manifestations, which significantly influence disease progression and quality of life. Diagnosis remains challenging and is based on clinical, endoscopic, and histological criteria [1,2]. Between 5% and 15% of patients exhibit overlapping features of both diseases and are classified as having indeterminate colitis (IC) [1].

IBD is an evolving global health issue, with increasing incidence in developing countries [3] and considerable morbidity in Western nations [4].

Although its etiology is not fully understood, several factors have been implicated, including genetic predisposition, gut microbiota alterations, dysregulated immune responses [5], and environmental influences [3]. A positive family history is the best-documented risk factor, with about 15% of affected individuals having a first-degree relative with the disease [1].

The pathogenic mechanisms underlying IBD also contribute to disease-related complications, which occur in up to 50% of cases. These include intestinal and systemic manifestations such as cutaneous, ocular, musculoskeletal, pulmonary, renal, thromboembolic, and hepatobiliary disorders. Hepatobiliary involvement ranges from mild, asymptomatic liver enzyme elevations to severe conditions such as primary sclerosing cholangitis or cholangiocarcinoma [6]. Other liver diseases linked to IBD include autoimmune hepatitis, gallstones and non-alcoholic fatty liver disease—recently redefined as metabolic dysfunction-associated steatotic liver disease (MASLD) [7,8].

MASLD is now the most prevalent liver disorder worldwide, affecting up to 30% of the population [9]. It is characterized by hepatic fat accumulation in the absence of significant alcohol intake (<20 g/day for women, <30 g/day for men) [10]. Although simple steatosis carries a low risk of progression, untreated cases may evolve into metabolic dysfunction-associated steatohepatitis (MASH), which markedly increases the risk of cirrhosis, hepatocellular carcinoma or liver failure [11,12]. The main risk factors for MASLD include metabolic syndrome, insulin resistance, hypertension [10], genetic variants such as the isoleucine to methionine substitution at position 148 in the patatin-like phospholipase domain containing 3 protein (PNPLA3-I148M) [9], and lifestyle habits including Western diets and physical inactivity [13].

Reported MASLD prevalence among IBD patients varies widely, likely due to methodological differences in diagnosing hepatic steatosis [2]. Recent studies using magnetic resonance imaging (MRI) or elastography have reported high MASLD rates in IBD populations—54.6% by MRI and 32.8% by elastography [14,15]. Several mechanisms may explain this association: chronic systemic inflammation, medication-induced hepatotoxicity, gut–liver axis dysfunction, and characteristic microbiota alterations observed in IBD [16,17,18,19].

**Figure 1:**
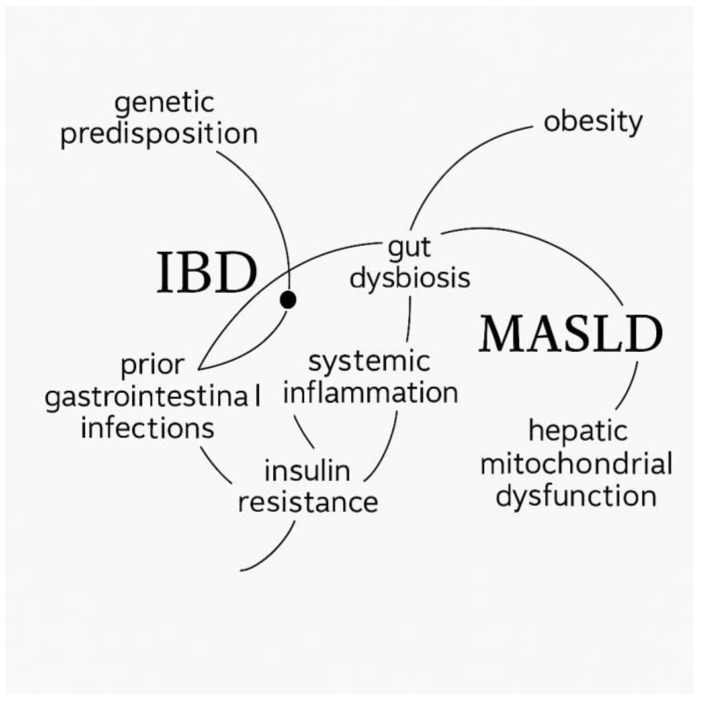
Conceptual illustration of the shared pathophysiological mechanisms linking inflammatory bowel disease (IBD) and metabolic dysfunction-associated steatotic liver disease (MASLD), generated using AI-assisted software (ChatGPT, OpenAI, GPT-5 model) based on data from reference [20].

Common risk factors such as genetic susceptibility, obesity, insulin resistance, and unhealthy lifestyle patterns also play a key role in the interplay between IBD and MASLD. Based on these mechanisms described in the literature [20], we generated a conceptual illustration (Figure 1) using artificial intelligence–assisted software to visualize the shared pathophysiological pathways connecting the two diseases.

MASLD is one of the most common liver conditions in patients with IBD, affecting nearly 30% of patients. MASH is the leading cause of elevated liver enzymes in IBD patients. Identifying these patients is crucial, not only to prevent hepatic disease progression but also to manage cardiovascular risk factors. This is particularly important because IBD itself increases cardiovascular risk [21,22,23].

The primary aim of this study was to assess hepatic risk—specifically MASLD—in patients with IBD by identifying risk factors, exploring links between these digestive disorders, and analyzing correlations between IBD characteristics (Harvey–Bradshaw Index (HBI), Total Mayo Score, C-reactive protein (CRP), fecal calprotectin) and MASLD features (steatosis and fibrosis severity). Secondary objectives include assessing the correlation between uric acid/high-density lipoprotein cholesterol ratio (UHRatio) and steatosis severity, as well as between non-invasive fibrosis scores like Fibrosis-4 (FIB-4), AST-to-Platelet Ratio Index (APRI), and actual fibrosis severity. Achieving these objectives will contribute to elucidating IBD complexity, its potential systemic impacts, and implications for clinical practice.

## 2. Results

The study included 58 patients, comprising 31 males (53.4%) and 27 females (46.6%), aged between 18 and 75 years, with a mean age of 41.88 years (±13.29 years). The average age at diagnosis was 35.90 years, ranging from 15 to 70 years, reflecting the bimodal distribution typically observed in IBD onset (15–30 years and over 60 years). The mean disease duration was 5.98 years, with the modal disease duration being two years. Most patients (n = 49, 84.5%) resided in urban areas, and 25 patients (43.1%) had completed higher education. Analysis of smoking status indicated that most patients were non-smokers (n = 31, 53.4%) or had quit smoking over one year prior (n = 17, 29.3%). The mean body mass index (BMI) was 23.78 kg/m^2^, indicating most patients were within the normal weight range.

Regarding disease types, of the 58 enrolled patients, 33 (56.9%) had CD, 24 (41.4%) had UC, and 1 patient was initially diagnosed with IC, but for statistical reasons, we included that patient in UC group. In CD patients, ileocolonic localization was the most frequent, observed in 21 patients (36.2%). The predominant phenotypes of CD included inflammatory (13 patients, 22.4%), stricturing (10 patients, 17.2%), penetrating (7 patients, 12.1%) forms and 3 patients presented overlapping forms. Specifically, 1 patient (1.72%) had both stricturing and perianal disease and 2 (3.45%) patients exhibited penetrating and perianal phenotypes. Among UC patients, most had pancolonic involvement (13 patients, 22.4%), followed by left-sided colitis (9 patients, 15.5%) and proctitis (3 patients, 5.2%). Treatment analysis showed that the majority of patients (n = 26, 44.82%) received biological therapies (Ustekinumab and Vedolizumab), 39.65% of patients received anti-TNF therapy, 19% were treated with aminosalicylates, 10% with corticosteroids, and only 2% with immunosuppressants. Surgical resection of gastrointestinal tract segments was performed in 10 out of the 58 patients (17.2%).

To identify risk factors potentially influencing MASLD development among patients diagnosed with IBD, statistical associations between IBD type, HBI, Mayo Score, CRP levels, fecal calprotectin, and the presence of hepatic steatosis/fibrosis were examined. Concerning steatosis distribution by IBD type, most CD patients (n = 26, 78.8%) and UC patients (n = 22, 91.7%) had no steatosis (grade S0, Controlled Attenuation Parameter (CAP) < 294 dB/m). A similar result was noted for the single patient diagnosed with IC.

A chi-square test was conducted, yielding a *p*-value of 0.703, indicating no statistically significant association between IBD type and steatosis grade. Given this result and the presence of only 1S patient diagnosed with IC among the 58 enrolled, this patient was subsequently categorized within the UC group (endoscopic evidence supported this decision). Consequently, subsequent statistical analyses were performed considering only two groups of IBD (CD and UC). Reapplication of the chi-square test following this adjustment still revealed no statistically significant result (*p*-value = 0.346).

Regarding fibrosis distribution by IBD type, all CD patients exhibited fibrosis grades F0-F1, corresponding to E < 8.2 Kilopascal (kPa). A similar pattern was observed among UC patients, with only one patient exhibiting an F1 fibrosis grade (E > 8.2 kPa). The patient initially diagnosed with IC presented fibrosis grade F0–F1. A chi-square test conducted with two disease categories showed no significant statistical association between fibrosis grade and IBD type (*p* = 0.246).

Further analyses were performed to evaluate associations between the HBI and hepatic steatosis grade, as well as between HBI and hepatic fibrosis grade. A significant statistical association was observed between the HBI and hepatic steatosis (***p* = 0.037**). However, no significant association was found with hepatic fibrosis (***p* = 0.854**).

The Total Mayo Score, used to evaluate UC severity, did not show a statistically significant association with hepatic steatosis (***p* = 0.839**). However, a marginal statistical significance at a **94%** confidence interval (***p*-value = 0.06**) was noted for hepatic fibrosis.

Regarding inflammatory syndrome in our patients, we analyzed the CRP and fecal calprotectin levels, as well as the grade of anemia. In the cohort of patients with CD, the median CRP level was 0.56 mg/dL (IQR [0.20–1.64]), whereas in patients with UC, the median was 0.20 mg/dL (IQR [0.20–1.19]). CD patients had a median hemoglobin level of 13.4 g/dL (IQR [12.5–14.35]). UC patients showed a median fecal calprotectin level of 60 µg/g (IQR [22–500]).

To investigate associations between inflammatory syndrome (represented by CRP levels) and hepatic steatosis and fibrosis grades, non-parametric tests (Kruskal–Wallis) were applied as follows (Table 1).

This association was not statistically significant (*p* = 0.247). Subsequently, the bootstrapping method—a statistical resampling technique useful for small datasets, generating new datasets through resampling with replacement from the original dataset—was employed. Using this method, a statistically significant association was identified between inflammation and fibrosis grades (***p* = 0.016**, Table 2). Similar statistically significant results were also observed regarding the association between fecal calprotectin levels and fibrosis (***p* = 0.022**, Table 3).

Given that the association results for steatosis grades suggested a non-normal distribution of this parameter, the variable was recoded into a new binary variable, “SteatosisGradenew,” with two possible values: 0 = patient without steatosis, 1 = patient with steatosis (regardless of degree). Subsequent tests were performed using this newly defined variable for steatosis characterization.

One of the study’s objectives was to verify the hypothesis proposed in other studies suggesting that the UHRatio is a non-invasive marker that could enhance the predictive capacity for early-stage MASLD in IBD patients. The results of tests applied to the study population confirmed a statistically significant association between UHRatio and steatosis grade (***p*-value = 0.044**, Table 4).

Ultimately, the association between non-invasive liver fibrosis evaluation scores (FIB-4 and APRI) and the degree of fibrosis in the study population was tested. The results of these analyses indicated that there was no statistically significant association for either of the two scores: FIB-4 score (***p*-value = 0.473**) and APRI score (***p*-value = 0.874**).

## 3. Discussion

This prospective study included 58 patients with IBD. It described their demographic, clinical and biological characteristics and evaluated the prevalence of MASLD, a potential complication of IBD.

Although this topic has attracted considerable interest, published data remain inconsistent, emphasizing the need for studies with larger and more homogeneous cohorts.

The relationship between MASLD and IBD appears to differ between CD and UC in both pathogenesis and clinical context.

In CD, the higher systemic inflammatory burden, profound gut dysbiosis and malabsorption-related metabolic disturbances may contribute to hepatic steatosis through altered lipid metabolism and mitochondrial dysfunction [24]. Dysbiosis in CD increases secondary bile acids, which activate the TGR5–mTOR–OXPHOS pathway in ileal immune cells, promoting ileitis and impairing farnesoid X receptor signaling in the liver. This disruption alters bile acid homeostasis and lipid metabolism, predisposing to hepatic steatosis [25,26,27]. Concurrently, the reduction in short-chain fatty acid producing bacteria compromises intestinal barrier integrity, facilitating translocation of lipopolysaccharide and other microbial products to the liver [28,29]. These bacterial products activate Toll-like receptor 4-mediated inflammatory pathways in hepatocytes and Kupffer cells, inducing oxidative stress and lipid accumulation characteristic of MASLD [30].

In contrast, MASLD in UC is more frequently associated with metabolic comorbidities, corticosteroid exposure and hepatotoxic effects of long-term aminosalicylate therapy [31]. These disease-specific pathways emphasize the multifactorial nature of liver involvement in IBD.

In our study, most patients with CD (26 patients, 78.8%) showed no hepatic steatosis (S0 grade). A similar pattern was observed in UC group, where 22 patients (91.7%) also had S0 grade steatosis. These results are consistent with previous reports showing variable findings. Some studies have identified CD as an independent risk factor for MASLD [32,33,34] and a recent meta-analysis reported that 67% of IBD patients with MASLD had CD, compared with 32% with UC [35]. In contrast, other studies have found a higher MASLD prevalence among UC patients (up to 55%) than among those with CD (39.5%) [36].

The statistically significant association between the HBI and hepatic steatosis (*p* = 0.037) suggests systemic extraintestinal inflammation in CD might contribute to MASLD development, aligning with longitudinal data presented by Gizard et al. [35]. However, the association between the HBI and hepatic fibrosis was not statistically significant (*p* = 0.854).

Among patients with UC no significant association was observed between the Total Mayo Score and hepatic steatosis (*p* = 0.839). This result is consistent with a large prospective case–control study that found no difference in steatosis prevalence between UC patients in remission and those with mild disease activity according to the Mayo score [37]. However, a marginal significance (*p* = 0.06) suggested that disease activity might influence fibrosis severity, as also reported by Bessissow et al. [37].

Regarding hepatic fibrosis, all CD patients (33 patients, 56.9%) demonstrated fibrosis grades F0–F1, a finding consistent within the UC patient group as well. These results diverge from previous findings by Aggarwal et al. [36], who reported higher hepatic steatosis and fibrosis prevalence in CD patients, identifying CD as an independent risk factor based on liver biopsy assessments [38].

Significant associations were observed between inflammatory markers (CRP, *p* = 0.016 and fecal calprotectin, *p* = 0.022) and hepatic fibrosis. These findings suggest that greater inflammatory activity is linked to more severe fibrosis, supporting previous evidence that higher intestinal disease activity increases the risk of MASLD [39].

It should also be noted that all bootstrap analyses were conducted using 1000 iterations with BCa confidence interval estimation. Where multiple related tests were performed, Bonferroni-adjusted thresholds were applied to reduce the likelihood of false-positive findings. Despite these precautions, the results should still be interpreted as exploratory given the limited sample size.

Our study identified a significant association between the UHRatio and hepatic steatosis (*p* = 0.04) in IBD patients, validating previous hypotheses by Xie et al., proposing this ratio as a useful non-invasive predictor for early MASLD and potential therapeutic target [40].

Although UHR showed a significant association with hepatic steatosis, its diagnostic performance is inferior to imaging-based modalities such as MRI or FibroScan, which remain the gold standard for liver assessment. Therefore, the UHR should be considered primarily as an accessible, preliminary screening tool to identify individuals at higher risk who may require further imaging evaluation, rather than as a diagnostic alternative.

The non-invasive fibrosis scores (FIB-4 and APRI) showed no correlation with fibrosis severity assessed by FibroScan.

The absence of a statistically significant correlation between FIB-4/APRI scores and fibrosis severity in our cohort likely reflects the relatively young mean age, the short disease duration in our study population, and the low prevalence of advanced fibrosis among participants. We fully acknowledge that future studies with larger samples could verify this explanation through subgroup analyses stratified by disease duration or patient age, which might reveal differential trends in hepatic involvement over time. Moreover, incorporating additional non-invasive biomarkers, such as the Enhanced Liver Fibrosis (ELF) score, could further improve the assessment of hepatic fibrosis. Although our current dataset does not include the parameters required to calculate the ELF score (hyaluronic acid, procollagen III amino-terminal peptide (PIIINP), and tissue inhibitor of metalloproteinases-1 (TIMP-1)), future studies integrating this biomarker may provide a more precise evaluation of fibrotic changes in patients with IBD.

From a preventive perspective, early control of intestinal inflammation, monitoring of metabolic risk factors, minimizing hepatotoxic drug exposure, and regular non-invasive hepatic assessment may help reduce the risk of MASLD in this population [41,42]. Moreover, the use of a simple and accessible biomarker such as the UHRatio, as demonstrated in our study, could facilitate earlier detection of MASLD, enable better risk stratification for further imaging-based evaluation and support the timely implementation of preventive measures aimed at reversing or halting hepatic disease progression.

The interpretation of these results should take into account that, although cases of viral hepatitis and alcohol consumption were excluded from the study, drug-induced hepatotoxicity may still have influenced the findings. In our cohort, the majority of patients received biological therapies, which are generally well tolerated but have been occasionally associated with adverse hepatic effects, including drug-induced hepatitis and cholestatic liver injury. Immunomodulators may also contribute to hepatic injury, such as drug-induced hepatitis, cirrhosis, or steatohepatitis, while aminosalicylates can cause hepatocellular toxicity. In addition, corticosteroid therapy may promote hepatic steatosis and steatohepatitis through metabolic mechanisms [43]. Therefore, part of the hepatic alterations observed in our study might reflect treatment-related effects rather than metabolic dysfunction alone. Future studies should include detailed analyses of medication exposure, duration and cumulative dosage, as well as stratification by treatment type, to better delineate the impact of drug-induced hepatotoxicity on liver involvement in IBD patients.

### Limitations

Our study has several limitations that should be acknowledged. First, it was conducted in a single tertiary center and included a relatively small cohort of 58 patients, which limits the generalizability of the findings. The recruitment process was particularly challenging for patients with severe disease activity, and difficulties in obtaining informed consent may have introduced selection bias, potentially affecting the representativeness of the study population.

In addition, variations in disease management, treatment regimens and lifestyle factors among patients could have influenced the observed outcomes. The assessment of liver steatosis and fibrosis was based solely on FibroScan, which, despite being a validated non-invasive tool, remains subject to operator-dependent variability. The unavailability of other imaging modalities such as MRI, as well as the lack of histopathological confirmation, further limits the precision of hepatic characterization. Another limitation of our study is the absence of a formal analysis of the diagnostic performance of the UHRatio, such as sensitivity, specificity, and optimal cut-off determination through ROC analysis. Consequently, the potential clinical applicability of this biomarker remains to be validated in larger, prospective cohorts using imaging-based reference standards.

Moreover, potential selection bias cannot be excluded, as the clinical and biological features of the enrolled patients may not fully reflect those of the broader IBD population. Statistical interpretation was also constrained by the modest sample size and the clinical heterogeneity of the cohort.

Future multicenter studies involving a larger and more diverse IBD population across multiple institutions, ideally at a national level, are warranted to validate our findings and enhance their external validity.

## 4. Materials and Methods

This study is a prospective analysis involving 58 patients diagnosed with IBD, registered and treated at the Gastroenterology 1 Clinic of the Emergency University Hospital Bucharest, Romania, between September 2023 and May 2024. The study was conducted in accordance with the Declaration of Helsinki and approved by the Ethics Committee of Emergency University Hospital Bucharest, Romania; (number and date of the Ethics Committee approval are as follows: 30354/12 June 2024).

Inclusion criteria comprised patients aged over 18 years who provided written informed consent for biological testing (complete blood count, coagulation profile, blood biochemistry, fecal calprotectin) and transient elastography (Fibroscan). Exclusion criteria included age below 18 years, pregnancy, chronic alcohol consumption (>20 g/day for women, >30 g/day for men), known liver disease (chronic viral hepatitis, autoimmune hepatitis, hepatic neoplasia, etc.), bacterial intestinal infections (Clostridioides difficile, intestinal tuberculosis) and cognitive impairment preventing informed consent.

Demographic and clinical profiles were assessed to identify potential MASLD risk factors, collecting data including age, sex, weight, height, body mass index, living environment (rural/urban), education level (primary, secondary, high school, post-secondary, university), smoking status (patients who quit smoking less than one year prior were classified as smokers, those who quit over one year prior as non-smokers), personal medical history (previous intestinal resections related to IBD treatment), symptomatology, and physical examination findings.

Paraclinical assessments included Fibroscan and the following laboratory analyses: aspartate aminotransferase (AST), alanine aminotransferase (ALT), gamma-glutamyl transferase (GGT), alkaline phosphatase (AP), total bilirubin (TB), glucose, glycated hemoglobin (HbA1c), uric acid, total cholesterol high-density lipoprotein cholesterol (HDL-c), low-density lipoprotein cholesterol (LDL-c), triglycerides (TG), hemoglobin (Hb), platelets (PLT), fibrinogen, CRP, and fecal calprotectin.

Using laboratory results, non-invasive hepatic fibrosis scores, including FIB-4 (calculated based on age, AST and ALT values, and platelet count) and APRI (AST, AST reference values, platelet count), and the UHRatio—a recently evaluated parameter associated with MASLD—were calculated.

Transient elastography measurements of CAP and liver stiffness (E) were used to assign steatosis and fibrosis grades, respectively. Hepatic steatosis was considered present with mean CAP values >294 dB/m, while hepatic fibrosis was defined as E values >8.2 kPa.

Regarding IBD characterization, collected data included disease type, localization, and phenotype (in CD). Disease severity was evaluated using the HBI for CD (HBI <5: clinical remission, 5–7: mild, 8–16: moderate, >16: severe), and the Total Mayo Score for UC (Total Mayo Score: 1–2 clinical remission, 3–5 mild, 6–10 moderate, 11–12 severe).

Data management was performed using Microsoft Excel 2010 (Microsoft Office suite) and statistical analyses were conducted using IBM SPSS Statistics v27. Statistical methods included descriptive statistics, cross-tabulations, chi-square tests, non-parametric tests, variance analysis, and logistic and multiple regression analyses.

To address the small sample size and potential non-normality of the data, a bootstrap resampling procedure was applied for selected non-parametric tests. The analyses were performed in IBM SPSS Statistics v27 using 1000 resampling iterations with replacement. Confidence intervals and significance levels were estimated using the bias-corrected and accelerated (BCa) method, which offers improved accuracy for small samples. This approach ensured stability of *p*-values and robustness of the results. When multiple related comparisons were conducted (e.g., assessing associations between inflammatory markers and both steatosis and fibrosis), Bonferroni-adjusted significance thresholds were applied to mitigate the risk of type I error.

## 5. Conclusions

MASLD is a globally rising health issue, both as a standalone disease and in association with IBD. Our study demonstrated no statistically significant difference between IBD types and the occurrence of hepatic steatosis or fibrosis, suggesting neither CD nor UC independently serves as a hepatic complication risk factor. However, intestinal disease severity correlated significantly with steatosis prevalence in CD and fibrosis prevalence in UC. Non-invasive tests (FIB-4, APRI) did not correlate with fibrosis in our patient cohort. Routine screening for MASLD in IBD patients may improve early detection and management of liver related complications and also cardiovascular ones. Our study confirms that the UHRatio, a non-invasive tool, may enhance early MASLD prediction and could be used in screening in IBD patients.

Although currently debated extensively, the literature remains inconclusive regarding the intersection of these two conditions, underscoring the need for further research to develop effective preventive and therapeutic strategies.

## Figures and Tables

**Table 1:** Association between C-reactive protein (CRP) levels and steatosis grade.

Steatosis Grade	N	CRP (mg/dL)	Mean Rank	*p*-Value
Grade 1	49	4.23 ± 1.86	31.04	
Grade 3	1	2.15 ± –	17.50	
Grade 4	8	3.52 ± 1.47	21.56	
Total	58			0.247

Kruskal–Wallis H = 2.80; Monte Carlo significance = 0.268 (95% CI: 0.260–0.277). CRP, C-reactive protein.

**Table 2:** Association between inflammatory syndrome characterized by CRP levels and fibrosis grade.

Fibrosis Grade	N	CRP (mg/L, Mean ± SD)	*p*-Value
Grade 1	57	1.02 ± 1.34	
Grade 2	1	0.20 ± –	0.016

Bootstrap for Independent Samples Test, based on 613 samples.

**Table 3:** Association between fecal calprotectin levels and fibrosis grade.

Fibrosis Grade	N	Fecal Calprotectin (µg/g, Mean ± SD)	*p*-Value
Grade 1	57	572.47 ± 954.99	
Grade 2	1	22.00 ± –	0.022

Bootstrap for Independent Samples Test, based on 645 samples.

**Table 4:** Association between uric acid/HDL-cholesterol ratio (UHRatio) and steatosis grade (Mann–Whitney U test).

Steatosis Grade	N	UHRatio (Mean Rank)	Sum of Ranks	*p*-Value
Grade 0	49	27.84	1364.00	
Grade 1	9	38.56	347.00	0.044
Total	58			

Test: Mann–Whitney U; Monte Carlo significance (1-tailed) = 0.044 (95% CI: 0.040–0.048); Monte Carlo significance (2-tailed) = 0.086 (95% CI: 0.081–0.092). UHRatio, uric acid-to-HDL-cholesterol ratio.

## Data Availability

The raw data supporting the conclusions of this article will be made available by the authors on request.

## Abbreviations

The following abbreviations are used in this manuscript:

ALT	Alanine aminotransferase
AP	Alkaline phosphatase
APRI	AST-to-platelet ratio index
AST	Aspartate aminotransferase
BMI	Body mass index
CAP	Controlled attenuation parameter
CD	Crohn’s disease
CRP	C-reactive protein
FIB-4	Fibrosis-4 score
GGT	Gamma-glutamyl transferase
Hb	Hemoglobin
HbA1c	Glycated hemoglobin
HDL	High-density lipoprotein cholesterol
HBI	Harvey–Bradshaw Index
IBD	Inflammatory bowel disease
IC	Indeterminate colitis
kPa	Kilopascal
LDL-c	Low-density lipoprotein cholesterol
MASLD	Metabolic dysfunction-associated steatotic liver disease
MASH	Metabolic dysfunction-associated steatohepatitis
MRI	Magnetic resonance imaging
PIIINP	Procollagen III amino-terminal peptide
PLT	Platelets
PNPLA3-I148M	Isoleucine-to-methionine substitution at position 148 in the patatin-like phospholipase domain containing 3 protein
TB	Total bilirubin
TG	Triglycerides
TIMP-1	Tissue inhibitor of metalloproteinases-1
UC	Ulcerative colitis
UHRatio	Uric acid-to-HDL cholesterol ratio
